# Molecular techniques in haematopathology: what and how?

**DOI:** 10.1111/his.15332

**Published:** 2024-10-15

**Authors:** Gaurav Chatterjee, Rong He, Nikhil Patkar, David Viswanatha, Anton W Langerak

**Affiliations:** ^1^ Hematopathology Department, ACTREC Tata Memorial Centre Mumbai India; ^2^ Division of Hematopathology Mayo Clinic Rochester MN USA; ^3^ Laboratory Medical Immunology, Department of Immunology Erasmus MC Rotterdam the Netherlands

## Abstract

Here we review the ‘what and how’ of molecular techniques used in the context of haematopathological diagnostics of both lymphoid and myeloid neoplasms. Keeping in mind that the required resources for molecular testing are not universally available, we will not only discuss novel and emerging techniques that allow more high‐throughput and sophisticated analyses of lymphoid and myeloid neoplasms, but also the more classical, low‐cost alternatives and even some workarounds for molecular testing approaches. In this review we also address other key aspects around molecular techniques for haematopatholgy diagnostics, including preanalytics, data interpretation, and data management, bioinformatics, and interlaboratory precision and performance evaluation.

## Graphical abstract

Schematic representation of various molecular alterations relevant to lymphoid and myeloid neoplasms and the different techniques that can be used for detection.

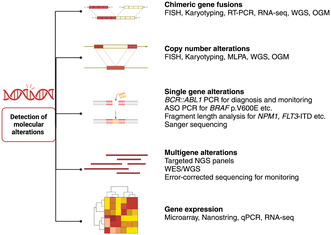

**Abbreviations**
AI: artificial intelligence
BM: bone marrow
CCUS: clonal cytopenia of undetermined significance
CH: clonal hematopoiesis
CHIP: CH of indeterminate potential
CLSI: Clinical and Laboratory Standards Institute
CMA: chromosome micro array
cnLOH: copy neutral loss of heterozygosity
CNV: copy number variation
ddCPR: droplet digital PCR
ELN: European Leukemia Net
ERIC: European Research Initiative on CLL
FISH: fluorescence in situ hybridization
GATK: Genome Analysis Toolkit
IG: immunoglobulin
LDT: lab‐developed test
MRD: measurable residual disease
NCCN: National Comprehensive Cancer Network
NGS: next generation sequencing
NOS: not otherwise specified
OGM: optical genome mapping
PB: peripheral blood
qPCR: quantitaive PCR
SNV: small nucleotide variant
SV: structural variant
TAT: turn‐around time
TR: T‐cell receptor
VAF: variant allele frequency
WES: whole exome sequencing
WGS: whole genome sequencing

## Background

Diagnosis and classification of haematological malignancies has traditionally relied on cyto‐ and/or histomorphology and immunophenotyping to define cell lineage and maturation stage, in combination with observed clinical features that accompany the malignancy (WHO HAEM 3 and 4 books).^1^, ^2^ Biological features were initially underrepresented, mostly because these were simply not known or not very well described. This has notably changed with the rapidly growing number of identified genomic aberrations that disclose different biological (sub)entities in both lymphoid and myeloid neoplasms. In fact, genomic aberrations, that is fusions, structural variants (SVs), but also single‐nucleotide variations (SNVs), have increasingly become so important that their detection through molecular diagnostics has gained a dominant role in classification next to morphology, phenotyping, and clinical characteristics. This is particularly true for the most recent update of the classification: the 5th edition of the World Health Organization Classification of Haematolymphoid tumours (WHO‐HAEM5) as well as the International Consensus Classification on Lymphoid and Myeloid Neoplasms (ICC), both of which heavily rely on molecular diagnostic criteria.^3^, ^4^, ^5^, ^6^ With the growing spectrum of common and differentiating molecular and genetic abnormalities, much more information has become available, which holds promise for the individual patient, as it paves the way to precision medicine.

**Figure 1:**
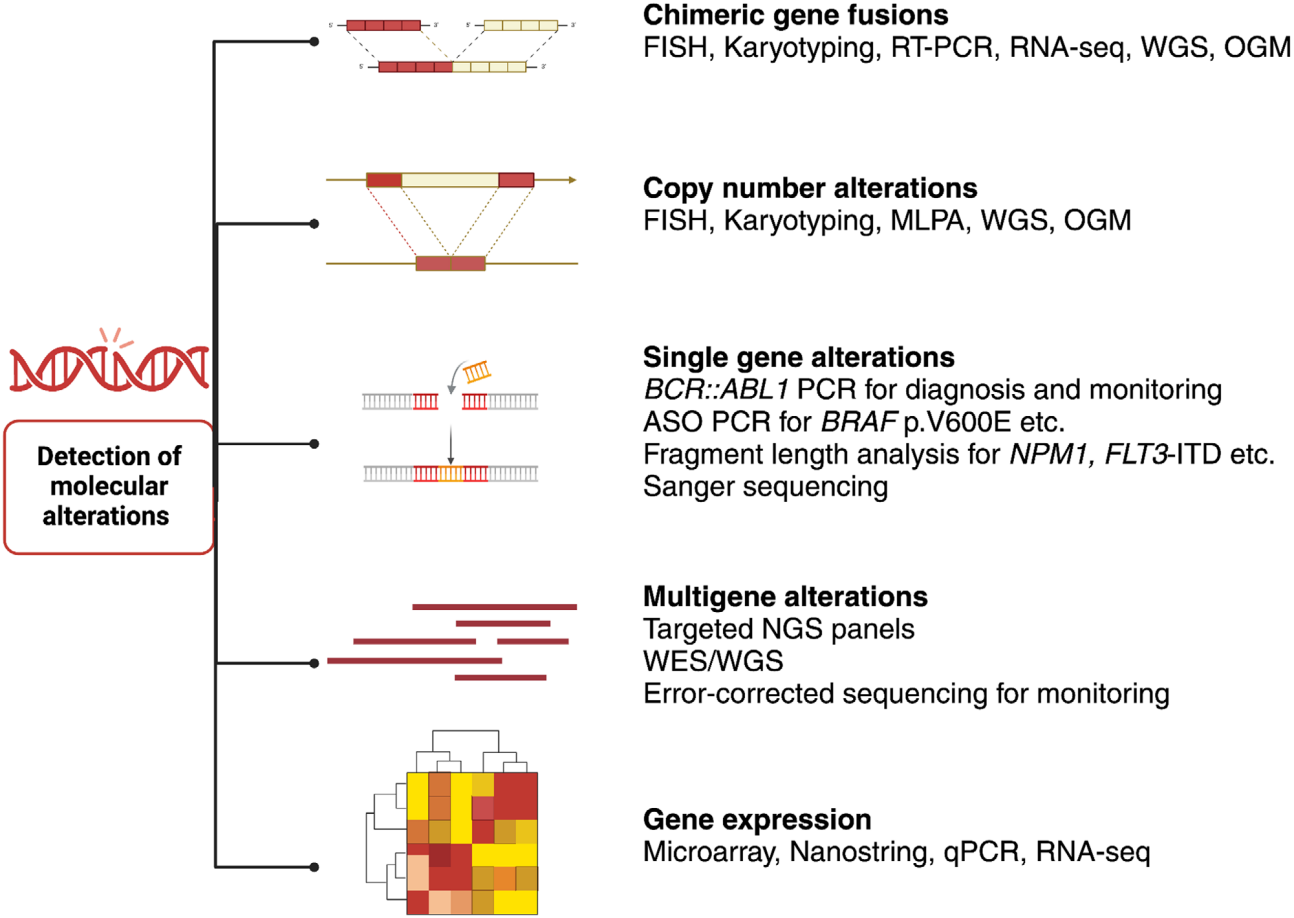
Schematic representation of various molecular alterations relevant to lymphoid and myeloid neoplasms and detection techniques. Sensitivity levels vary depending on the techniques used. For example, Sanger sequencing is unable to detect subclonal (<20%) single‐nucleotide changes, sequencing‐based approaches, and karyotyping may miss subclonal copy number alterations. A few selected examples of single‐gene tests are shown in the figure. Created with BioRender.com. ASO PCR, allele‐specific oligonucleotide PCR; MLPA, multiplex ligation‐dependent probe amplification; OGM, optical genome mapping; qPCR, quantitative/real‐time PCR; WGS, whole genome sequencing.

Here we review the ‘what and how’ of molecular techniques used in the context of haematopathological diagnostics of both lymphoid and myeloid neoplasms. Keeping in mind that the required resources for molecular testing are not universally available, we not only discuss novel and emerging techniques that allow more high‐throughput and sophisticated analyses of lymphoid and myeloid neoplasms, but also the more classical, low‐cost alternatives and workarounds for molecular testing approaches. Among the low‐throughput molecular approaches are (quantitative) polymerase chain reaction (PCR)‐based detection (for gene fusions), droplet digital PCR (ddPCR) or classical Sanger sequencing (for hotspot variants), fragment analysis of fluorescently labelled PCR amplicons (for tandem duplications, or rearranged immunoglobulin [IG] and/or T‐cell receptor [TR] genes). Novel approaches mostly concern high‐throughput technologies and typically relate to next‐generation sequencing (NGS) protocols, using targeted gene panels for amplicon‐based templates or probe sets for capture hybridization of relevant gene sets. Unbiased high‐throughput approaches include whole‐exome sequencing (WES), whole‐genome sequencing (WGS), or RNA sequencing approaches that are being introduced to cover genome‐wide aberrations or whole transcriptome analysis, respectively. Targeted NGS or even WES/WGS approaches can also be employed for the complex genetics of IG/TR rearrangements. Even more advanced sequencing technologies concern long‐read sequencing and single‐cell sequencing. In Table 1 and Figure 1, the most relevant features of all these technologies are summarized. Finally, we will also address aspects of preanalytics, data interpretation and data management, bioinformatics, and interlaboratory precision and performance evaluation, also touching upon quality systems and reference labs for certain molecular tests.

**TABLE 1:** Overview of different molecular technologies employed in haematopathology diagnostics

Technology	Principle	Application/example
*Low‐throughput*		
PCR	PCR‐based amplification of gDNA/ cDNA target; qualitative scoring of endpoint measurement	Fusion transcripts, fusion genes
qPCR (quantitative real‐time PCR)	Real‐time PCR‐based amplification of gDNA/cDNA target; quantitative scoring	Fusion transcripts, fusion genes; IG/TR rearrangements
ddPCR (droplet digital PCR)	PCR‐based amplification of gDNA target/genetic variant in sequestered templates; digital, quantitative scoring of endpoint measurement	Fusion transcripts, fusion genes; (onco)genetic variants (esp. hotspots)
Fragment analysis (*GeneScan analysis*)	High resolution fragment size analysis of PCR‐based amplicons; fluorescent intensity scoring	Insertions and deletions; duplications or repeats; IG/TR rearrangements
Sanger sequencing	Bulk sequencing of PCR‐generated amplicons	(Onco)genetic variants
FISH analysis	Fluorescently labelled probe‐based detection of chromosome regions or centromeres	Fusion genes, chromosome aberrations, duplications, deletions, numerical chromosome aberrations
*High‐throughput*		
Amplicon‐based NGS	Parallel sequencing of library of PCR‐generated templates	Fusion genes; IG/TR rearrangements; (onco)genetic variants
Capture‐based NGS	Parallel sequencing of library of capture hybrid‐generated templates	Fusion genes; IG/TR rearrangements; (onco)genetic variants
WES	Parallel sequencing of library of exome‐based templates	Genome‐wide aberrations (coding regions)
WGS	Parallel sequencing of library of genome‐based templates	Genome‐wide aberrations
RNA sequencing	Parallel sequencing of all transcripts (transcriptome)	Transcriptome analysis (levels, fusion transcripts, alternative splicing)
Long‐read sequencing	Sequencing of long templates	IG/TR rearrangements
Molecule‐based sequencing	Molecule‐based parallel sequencing of library with barcoded unique molecular identifiers (UMIs)	Measurable residual disease quantitation
Single cell RNA sequencing	Single cell sequencing of all transcripts (barcode‐labelled cells)	Transcriptome analysis (levels, fusion transcripts, alternative splicing) on a per cell basis

cDNA, copy DNA; FISH, fluorescent *in situ* hybridization; gDNA, genomic DNA; IG, immunoglobulin; NGS, next‐generation sequencing; TR, T‐cell receptor.

## Preanalytics as a key aspect of accurate molecular testing

Prior to any further description of the use of molecular techniques, it should be stressed that the actual added value of these techniques in lymphoid and myeloid neoplasms critically depends on preanalytical aspects. This entails the choice for the type of material, the sample collection, preservation, and transport. Although blood is logically the most easily accessible compartment, it may not always be the preferred sample type. This holds for more localized tumours in which case bone marrow aspirates, bone marrow biopsies, lymph node biopsies, or even tumour biopsies might be more informative. Similarly, monitoring is only feasible using material in which residual cells can still be expected, or from which circulating tumour DNA can be obtained. With respect to biopsies, collection in formalin might block certain molecular approaches for which the DNA or RNA should be less fragmented or better preserved. Finally, it is important to realize that good preservation of body fluids (cerebrospinal fluid, eye fluid, broncho‐alveolar lavage) with serum or fixatives allows better cell survival for cellular testing, and thus also increases the yield for molecular testing and its sensitivity.

## Rationale for use of molecular techniques in lymphoid and myeloid neoplasms

Underlying molecular abnormalities define or play a pivotal role in establishing the diagnosis of various lymphoid and myeloid neoplasms as defining or supportive molecular markers (Tables 2 and 3). In addition, molecular aberrations also help to improve risk‐stratification and prediction, and even define biology‐driven neoplasm subgroups with specific clinical behaviour that may profit from targeted treatment.^3^, ^5^

**TABLE 2:** Established diagnostic, prognostic, and/or predictive molecular markers for particular types of lymphoid neoplasms*

Type of lymphoid neoplasm	Gene aberration or variant	Main detection methods	Implication
B‐cell lymphoblastic leukaemia	Hyperploidy, hypoploidy	FISH, cytogenetics	Diagnostic, prognostic
	Gene fusions, such as *BCR::ABL1*, *ETV6::RUNX1*, *TCF3::PBX1*, *TCF3::HLF*, *IGH::IL‐3*, *KMT2A*	FISH, qPCR, NGS	Diagnostic, prognostic
	*BCR::ABL1*‐like features, *ETV6::RUNX1*‐like features	Gene expression (RNA seq)	Diagnostic, prognostic
Chronic lymphocytic leukaemia (CLL)	Chromosome aberrations, such as del17p, del11q, del13q, trisomy 12	FISH, cytogenetics, NGS (capture)	Prognostic
	*TP53* mutation	Sanger sequencing, NGS	Prognostic, predictive
	IGHV somatic mutation status	Sanger sequencing, NGS	Prognostic, predictive
	BcR stereotyped subset (#2, #8)	Sanger sequencing, NGS	Prognostic
Hairy cell leukaemia (HCL)	*BRAF* V600E mutation	Sequencing, ddPCR	Diagnostic
Lymphoplasmacytic lymphoma (LPL)	*MYD88* L265P mutation	Sequencing, ddPCR	Diagnostic
	*CXCR4* mutation		
Multiple myeloma (MM)	Translocations, such as those involving *CCND*, *MAF*, *NSD2*	FISH, cytogenetics, NGS	Diagnostic
	Hyperdiploidy	FISH, cytogenetics	Diagnostic
Nodal marginal zone lymphoma (nMZL)	*KMT2D*, *PTPRD*, *NOTCH2*, *KLF2* mutation	Sanger sequencing, NGS	Diagnostic
Extranodal marginal zone lymphoma (eMZL) (gastric, pulmonary)	*BIRC3::MALT1* fusion	qPCR, FISH, cytogenetics, NGS	Diagnostic
Extranodal marginal zone lymphoma (eMZL) (ocular)	*TNFAIP3* mutation	Sanger sequencing, NGS	Diagnostic
Extranodal marginal zone lymphoma (eMZL) (salivary)	*GPR34*, *TNFRSF14*, *CD274*, *TET2* mutation	Sanger sequencing, NGS	Diagnostic
Follicular lymphoma (FL)	*IGH::BCL2* aberration	qPCR, FISH, cytogenetics, NGS	Diagnostic
Mantle cell lymphoma (MCL)	*IGH::BCL1* aberration	qPCR, FISH, cytogenetics, NGS	Diagnostic
Burkitt lymphoma (BL)	*IGH::MYC* aberration	qPCR, FISH, cytogenetics, NGS	Diagnostic
High‐grade B‐cell lymphoma	*MYC* rearrangement plus *BCL2* rearrangement	qPCR, FISH, cytogenetics, NGS	Diagnostic, prognostic
Large B‐cell lymphoma	11q aberration	FISH, cytogenetics	Diagnostic, prognostic
	*IRF4 rearrangement*	FISH, cytogenetics	Diagnostic, prognostic
Diffuse large B‐cell lymphoma (DLBCL) (CNS, eye)	*MYD88* L265P, *CD79a* mutation	Sequencing, ddPCR	Diagnostic
T‐large granular lymphocyte leukaemia (T‐LGLL)	*STAT3* mutation (CD8 and TCRgd subtypes), *STAT5b* mutation (CD4 and CD8 subtypes)	Sanger sequencing, NGS	Prognostic
Anaplastic large cell lymphoma (ALCL)	*ALK* rearrangement	FISH, cytogenetics	Prognostic
Breast implant‐associated anaplastic large cell lymphoma (ALCL)	*STAT3*, *STAT5B*, *JAK1*, *JAK2*, *SOCS1*, *SOCS3* mutation	Sanger sequencing, NGS	Diagnostic
Follicular helper T‐cell lymphoma, angioimmunoblastic‐type	*RHO1*, *IDH2* mutation	Sanger sequencing, NGS	Diagnostic

* As defined in the WHO‐HAEM5 and/or ICC classification; see for further details and references on individual aberrations the respective WHO‐HAEM5 and ICC publications^3^, ^4^, ^5^, ^6^ and other reviews in this Annual Review Issue.

**TABLE 3:** Clinical relevance of molecular alterations and techniques in myeloid neoplasms

Myeloid neoplasm	Defining for subtypes	Helps in establishing diagnosis	Risk stratification/prognosis	Therapeutic opportunity	Techniques commonly used in evaluation
Myeloid neoplasmswith germline predisposition	Currently well‐recognized entities include germline P/LP variant of: *CEBPA* *DDX41* *TP53* *RUNX1* *ANKRD26* *ETV6* *GATA2* *SAMD9* *SAMD9L* Down syndrome Inherited BM failure syndromes RASopathies	Critical considerations include selection of patients and samples	Additional somatic mutations are associated with progression to myeloid neoplasms	Surveillance plan Planning and selection of donor for HSCT Future development of gene‐specific strategy	Targeted panel‐based DNA‐sequencing, augmented WES, CMA The NGS panel may need to be specifically tuned to detect all inherited myeloid malignancy related alterations including small deletions or promoter/intronic variants
CH and Clonal cytopenia	Common genes mutated in CHIP and CCUS are: *DNMT3A*, *TET2*, *ASXL1*, *JAK2*, *TP53*, *SF3B1*, *PPM1D*, *SRSF2*, *IDH1*, *IDH2*, *U2AF1*, *KRAS*, *NRAS*, *CTCF*, *CBL*, *GNB1*, *RUNX1*, *EZH2*, *PTPN11*, etc. VEXAS syndrome is characterized by somatic *UBA1* mutations	—	CH risk score (CHRS) that incorporates high‐risk mutations has been proposed to predict risk of developing myeloid neoplasms Mutations in *TP53*, *PPM1D*, *CHEK2* confer high risk of developing t‐ myeloid neoplasms in cancer patients with cytotoxic chemotherapy/radiotherapy	Yet to be defined. CHIP‐clinics can be considered for management	Targeted panel‐based DNA‐sequencing is commonly used Unbiased approaches such as WES/WGS and error‐corrected sequencing can be used based on the required trade‐off between sensitivity and cost
MDS	MDS with low blasts and del(5q) MDS with low blasts and SF3B1 mutation MDS with biallelic TP53 inactivation	Commonly genetic alterations in MDS include *TET2*, *ASXL1*, *SF3B1*, *DNMT3A*, *SRSF2*, del(5q), *RUNX1*, *TP53*, *STAG2*, complex karyotype, *U2AF1*, +8, *EZH2*, *ZRSR2*, *BCOR*, ‐Y, −7/del(7q), del(20q), *DDX41*, *SAMD9*, *SAMD9L*, etc.	IPSS‐M is an integrated prognostic model incorporating molecular alterations	Lenalidomide is standard of care in patients with anaemia, lower risk MDS and del(5q) TP53 mutations may predict inferior response to lenalidomide in del(5q) MPN	FISH, cytogenetics/CMA and targeted panel‐based DNA‐sequencing. In addition to mutations, detection of deletion/cnLOH of *TP53* by CMA/NGS is essential
MPN/MPN	MDS/MPN with *SF3B1* mutation and thrombocytosis	Common mutations include *TET2*, *SRSF2*, *ASXL1*, *RUNX1*, *CBL*, *SETBP1*, *ETNK1*, *DNMT3A*, *KRAS*, *NRAS*, *EZH2*, *SF3B1*, *U2AF1*, *ZRSR2*, *IDH1*, *IDH2*, *BCOR*, *NF1* etc.	Several risk‐scores incorporating molecular features have been proposed for CMML	—	FISH, cytogenetics/CMA and targeted panel‐based DNA‐sequencing
					
MPN	CML: *BCR::ABL1* CNL: *CSF3R* mutations JMML: RAS pathway gene mutations (*PTPN11*, *KRAS*, *NRAS*, *NF1*, *CBL*)	*JAK2*p.V617F, *CALR* and *MPL* mutations are commonly observed and helps in establishing diagnosis in PV, ET and PMF	Several risk scores incorporating structural changes and somatic mutations are proposed in PMF (MIPSS*v*2, GIPSS)	Several TKIs in CML *ABL1* kinase domain mutation analysis can guide in choice of TKI *JAK*‐inhibitors	FISH, cytogenetics/CMA, PCR and targeted panel‐based DNA‐sequencing qPCR/dPCR.for *BCR::ABL1* monitoring. Sanger sequencing/NGS for *ABL1* kinase domain mutation analysis in patients with CML *JAK2*p.V617F. *CALR* indels, *MPL*W515L/K mutations can be detected using PCR and capillary electrophoresis
Mastocytosis	*KIT*p.D816V and other *KIT* exon 17 mutations are seen in >90% of systemic mastocytosis (SM) and are part of diagnostic criteria	—	*SRSF2*, *ASXL1*, *RUNX1*, *NRAS* mutations adversely affect prognosis of SM	*KIT*D816V inhibitors	Sanger sequencing/NGS
Eosinophilia	MLN‐Eo are defined by following fusions: *PDGFRA*‐r *PDGFRB*‐r *FGFR1*‐r *JAK2‐*r *FLT3‐*r *ETV6::ABL1*	Mutations in *KIT*, *JAK2*, *STAT5B*, *JAK1* and *STAT5A* genes have been reported in clonal eosinophilia	—	Imatinib in *PDGFRA/B*‐r *FGFR1*‐inhibitors *JAK2*, *FLT3* and *ABL1* inhibitors	FISH, cytogenetics/CMA, PCR and targeted panel‐based DNA and RNA sequencing
AML	AML‐defining genomics includes fusions and *NPM1/CEBPA* or myelodysplasia‐related (AML‐MR) mutations	Other important genes commonly altered/described include: *FLT3‐*ITD, *FLT3‐*TKD, *IDH1*, *IDH2*, *KMT2A‐*PTD, *WT1*, *NRAS*, *KRAS*, *GATA1*, *DNMT3A*, *TET2*, *ASXL2*, *UBTF*‐TD	ELN 2022 risk stratification incorporates several genetic factors Separate risk‐scoring systems have been proposed for patients treated with HMA + VEN	FDA approved therapy: *PML::RARA* *FLT3* *IDH1* *IDH2* *BCR::ABL1* Other promising therapies: Menin inhibitors in *KMT2A*‐r and *NPM1* mutated AML	FISH, cytogenetics/CMA, PCR and targeted panel‐based DNA and RNA sequencing Recurrent common alterations such as *NPM1*, *FLT3*‐ITD, *CEBPA* etc. can be detected using PCR and capillary electrophoresis qPCR/dPCR and error‐corrected sequencing for MRD

CCUS, clonal cytopenia of undetermined significance; CH, clonal haematopoiesis; CHIP, clonal haematopoiesis of indeterminate potential; CMA, chromosomal microarray; CML, chronic myeloid leukaemia; CNL, chronic neutrophilic leukaemia; cnLOH, copy‐neutral loss of heterozygosity; dPCR, digital PCR; ET, essential thrombocythemia; HMA + VEN, hypomethylating agents and Venetoclax; JMML, juvenile myelomonocytic leukaemia; MLN‐Eo, myeloid/lymphoid neoplasm with eosinophilia; P/LP, pathogenic/likely pathogenic; PMF, primary myelofibrosis; PV, polycythemia vera; qPCR, quantitative/real‐time PCR; TKI, tyrosine kinase inhibitors; WES, whole‐exome sequencing.

### Diagnosis and classification of lymphoid neoplasms

Further classification of B‐lymphoblastic leukaemia/lymphoma entities is typically based on cytogenetic and/or molecular genetic abnormalities, most of which concern chromosomal ploidy changes and SVs of chromosomes. Traditionally, most of these chromosomal aberrations have been identified using fluorescence *in situ* hybridization (FISH) approaches (either as split‐signal FISH or fusion FISH), or through cDNA‐based PCR/quantitative PCR (qPCR) approaches. Especially, PCR approaches often suffer from false‐negativity in the case of scattered, uncommon, and newly identified breakpoint regions and/or newly identified partner genes, as in the case of *KMT2A* rearrangements that have a wide range of translocation partners. Hence, RNA sequencing (for SVs leading to fusion genes), WGS or NGS‐capture panels (for ploidy and SVs) have gained interest. Notably, other B‐lymphoblastic leukaemias/lymphomas are defined on the basis of genetic drivers that should be identified through gene expression or gene variant analysis, for which RNA sequencing and other high‐throughput NGS strategies are most suited. For T‐lymphoblastic leukaemias/lymphomas, molecular classification has not yet found its way into the latest classifications, but this is expected to change in a future update.

In mature B‐cell neoplasms, over the years molecular testing has allowed to more precisely (re)define certain entities. This is especially the case for chromosome aberrations involving the IG heavy chain (IGH) locus and one of the many partner genes, such as the genes *BCL1* (cyclin D1), *BCL2*, and *MYC*, which are defining for mantle cell lymphoma, follicular lymphoma, and Burkitt lymphoma, respectively. Within the family of large B‐cell lymphomas and also within multiple myeloma, biologically meaningful molecular entities have been distinguished based on different translocations. Traditionally, such chromosome aberrations have been detected by FISH or qPCR methods, with the latter being performed at the genomic DNA level, as no fusion transcripts are formed. Nowadays, capture NGS, WES, or WGS also allow identifying these aberrations. In parallel with the use of NGS for the detection of SVs, mutation detection as a support for lymphoma diagnosis has found its way into practice. Classification of particular mature B‐cell neoplasms can be confirmed by their specific SNVs, such as the *BRAF* V600E somatic mutation in >95% of hairy cell leukaemia (HCL) and the *MYD88* L265P mutation in >93%–97% of lymphoplasmacytic lymphoma (LPL) and in primary large B‐cell lymphoma in immune‐privileged sites. Although such hotspot mutations can be identified through sequencing (Sanger or NGS) methods, a sensitive and (relatively) affordable approach is ddPCR, which has shown its unique strength in supporting the diagnostic workup of vitreous fluid cytology samples to demonstrate primary vitreo‐retinal large B‐cell lymphoma.

Even though molecular genetic testing does not directly classify mature T‐/NK‐cell neoplasm entities, it may support diagnosing certain neoplasm types based on characteristic single‐nucleotide variants. This is the case for T‐large granular lymphocyte leukaemia (T‐LGLL) in which *STAT3* (CD8+ and TCRgd+ T‐LGLL subtypes) and *STAT5b* (CD4+ T‐LGLL subtype) mutations are frequent. Other frequently observed aberrations concern defining *ALK* rearrangements in ALCL, and more supportive *RHOA* and *IDH2* mutations in the follicular helper T‐cell lymphoma, angioimmunoblastic‐type. Finally, within peripheral T‐cell lymphoma Not otherwise specified, transcriptional programs might be used. NGS gene panels gain more importance in mature T‐/NK‐cell neoplasms, as many mutations are not restricted to hotspot regions. Additionally, gene expression profiles may help to classify entities or subtypes, partly as surrogates for immunophenotyping.

### Diagnosis and classification of myeloid neoplasms

In addition to qPCR for monitoring *BCR::ABL1*, *ABL1* kinase domain point mutation analysis provides important clinical information in predicting resistance and choosing appropriate tyrosine kinase inhibitor (TKI)/treatment strategies in chronic myeloid leukaemia (CML). As compared to Sanger sequencing, NGS provides higher sensitivity to detect clinically relevant low‐level and compound *ABL1* mutations.^7^, ^8^ Somatic mutations are identified in ~90% of patients with MDS or CMML, as opposed to cytogenetic alterations seen in ~40% of patients.^9^, ^10^, ^11^, ^12^ A significant proportion of acute myeloid leukaemia (AML) are now defined by underlying genomics, irrespective of blast percentage (or at a lower threshold of 10% as per ICC). Importantly, in addition to the MDS‐associated cytogenetics, mutations in chromatin modifiers (*ASXL1*, *BCOR*, *EZH2*), splicing‐cohesion complex genes (*SF3B1*, *SRSF2*, *U2AF1*, *ZRSR2*, *STAG2*) are significantly associated with AML progressing from MDS,^13^, ^14^ and are considered crucial for defining AML‐MDS related (AML‐MR). Due to all these changes, only ~5% of morphologically‐defined AMLs remain unclassified into any of the WHO5/ICC recognized genetic categories.^15^ In addition to biologic grouping, molecular alterations form the basis of risk‐stratifications in AML.^16^ Similarly, an integrated molecular prognostic model IPSS‐M has been recently developed in a large cohort of MDS/CMML patients.^9^ Finally, small molecule inhibitors of *FLT3*, *IDH1*, and *IDH2* are approved by the US Food and Drug Administration (FDA),^17^, ^18^, ^19^, ^20^ further establishing the clinical importance of testing of these genes.

Cytogenetics including FISH/karyotyping/CMA, and panel‐based (~75–100 genes) DNA‐sequencing is required for baseline characterization of a newly diagnosed myeloid neoplasms. PCR and capillary electrophoresis / fragment analysis can be used to rapidly detect *NPM1* and *FLT3‐*ITD (for AML), *JAK2* p.V617F, *CALR* indels, and *MPL* p.W515 (for MPN) alterations. Clinically relevant mutations such as *NPM1*, *FLT3*, *IDH1*, and *IDH2*, need to be reported in a time‐sensitive manner (~5 days). Targeted RNA‐sequencing can complement cytogenetic methods in the diagnosis of clinically important fusions, especially for eosinophilia‐associated fusions, cryptic fusions such as *ETV6::ABL1* and *NUP98*‐r, and for genes known to have multiple fusion partners, such as *KMT2A*‐r.^21^, ^22^, ^23^, ^24^, ^25^ Panel‐based or unbiased sequencing approaches can aid karyotyping/CMA in detecting focal/arm‐level copy number alterations, and allele‐specific copy number profiles.^26^, ^27^ Detection of copy loss or copy‐neutral loss of heterozygosity (cnLOH) of the *TP53* gene, along with mutations, is especially critical in patients with MDS, as only biallelic *TP53* inactivations (MDS‐biTP53) are associated with an inferior outcome and recognized as a distnict disease entity in WHO/ICC classifications. Detection of cnLOH is particularly challenging, as it requires SNP‐array, panel‐based sequencing incorporating genome‐wide SNP probes or WES/WGS. A *TP53* mutation with >49% Variant allele frequency (VAF) has been proposed to be a presumptive evidence of MDS‐biTP53 when germline variants are ruled out. Finally, WGS has shown promise as a potential one‐stop solution to detect all relevant genetic abnormalities in myeloid neoplasms.^27^, ^28^

Through widespread use of NGS, it has been recognized that some myeloid neoplasms are associated with deleterious germline genetic variants. In addition, mutated haematopoietic cell clones can be identified in individuals with advancing age but without evidence of a haematologic neoplasm, termed clonal haematopoiesis (CH).^28^, ^29^, ^30^ CH can occur with mutations in myeloid neoplasm‐associated genes at a VAF ≥2%, or with non‐MDS defining cytogenetic alterations, in either case termed CH of indeterminate potential^31^, ^32^ (CHIP). If clonal genetic alterations are associated with persistent (≥4 months) cytopenia(s) and the absence of definitive morphologic features of a myeloid neoplasm, this situation is referred to as clonal cytopenia of undetermined significance (CCUS).^33^ VEXAS syndrome is a recently recognized CH‐related condition that is characterized by progressive systemic autoinflammatory manifestations, vacuoles in myeloid/erythroid precursors in the bone marrow, and somatic mutations in the X‐linked gene *UBA1*. CHIP/CCUS clones can be observed when using several NGS‐based methods such as targeted gene panels, error‐corrected sequencing, WES, and WGS, at various respective levels of sensitivity.^33^ CHIP is by definition incidentally identified, but carries risks for subsequent development of myeloid neoplasms (e.g. MDS, AML), as well as nonneoplastic morbidities (e.g. cardiovascular events); however, the magnitude of risk is dependent on multiple factors, such as the specific gene mutation type, VAF, or prior exposure to DNA‐damaging agents (e.g. solid tumour treatment). CCUS is more significantly associated with myeloid neoplasm risk, particularly with key genetic alterations (e.g. RNA splicing genes, TP53, IDH1/2, etc.). CHIP and CCUS may also be “unmasked” following therapy in patients with established myeloid or lymphoid malignancies, manifesting as a persistent residual clonal population. Notably, clonal haematopoiesis following autologous or allogeneic stem cell transplantation has been associated with increased risks of therapy‐related myeloid neoplasms and poor engraftment, respectively. The identification of CHIP, and especially CCUS, requires greater scrutiny of the patient to assess for additional adverse clinical features, as well as closer monitoring to detect evolving myeloid neoplasia and nonhaematologic diseases.

### Clonality assessment for B‐ and T‐cell lymphoid proliferations

**Figure 2:**
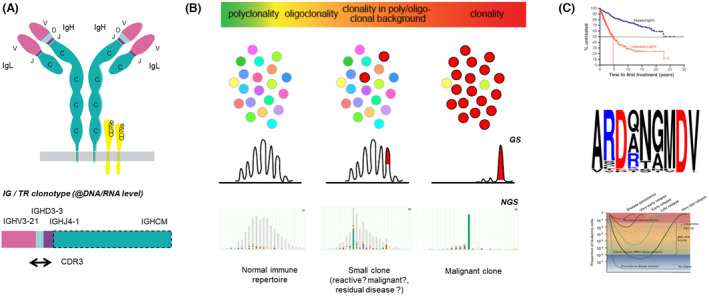
IG/TR rearrangement analysis and applications. (**A**) B and T cells carry highly specific antigen receptors, termed immunoglobulin (IG) and T‐cell receptors (TR), respectively. These antigen receptors, here exemplified for the immunoglobulin, consist of unique variable domains (purple colour variants) created by genetic rearrangements of V, D, and J genes on a per‐cell basis. The resulting V(D)J rearrangement is referred to as a clonotype that functions as a molecular fingerprint, especially through its CDR3 region. (**B**) IG/TR gene analysis of lymphoid cells discloses the heterogeneity of lymphoid cells, with a spectrum ranging from completely diverse (polyclonality) to fully identical (clonality). Clonality assessment can be done by fragment/Gene Scan (GS) analysis or through NGS methods. (**C**) IG/TR gene analysis can also be applied for prognosis in CLL through identification of somatically mutated vs unmutated cases (upper graphic) or even stereotypic subsets with a characteristic CDR3 (middle graphic). Another application concerns the analysis of IG/TR clonotypes for MRD purposes (lower graphic).

Additionally, one of the common challenges of diagnosing lymphoid proliferations is the distinction between hyperplastic lymphoproliferative disease and lymphoid malignancy, as this heavily impacts the choice of therapy. Even though this distinction can often be made through morphology and phenotyping, in an estimated fraction of 10%–15% of cases molecular clonality assessment is an important supportive methodology for this distinction.^34^ In theory, any somatically acquired genetic aberration could serve as a clonal marker, and thus as an indication of malignancy. That said, lymphoid cells are unique in the sense that they all harbour rearranged IG and/or TR genes that are formed through a combination of variable (V), diversity (D), and joining (J) genes. These rearrangements can be exploited as uniform markers for clonality assessment (Figure 2). In the light of the huge diversity of an estimated 10^12^ different IG/TR gene rearrangements in healthy B and T cells, respectively, the clear dominance of particular IG/TR antigen receptor rearrangements is to be interpreted as a sign of a clonal cell population that could support the diagnosis of a lymphoid malignancy. Notably, a word of caution is needed, as fulminant immune reactions, as in autoimmune and inflammatory settings and in immune reconstitution after chemo‐ and various forms of immunotherapy, may also give rise to clones of (activated) B or T cells.^35^ Additionally, IGH V gene (IGHV) somatic hypermutation status has since long been used as a valuable prognostic marker in chronic lymphocytic leukaemia (CLL) based on the 98% IGHV gene identity threshold (Figure 2). Initially, this threshold was determined through Sanger sequencing, but also NGS‐based protocols have been successfully introduced.

## Technological considerations and further technological advances

The genetic landscape of lymphoid and myeloid neoplasms is broad, featuring a wide array of clinically relevant variants. These range from small, single‐nucleotide aberrations to large chromosomal‐level SVs and CNV in the megabase range. Given the inherent advantages and limitations of each testing platform, the current standard of care for molecular genetic testing of haematologic neoplasms typically involves a combination of cytogenetic analysis (karyotyping and/or FISH) to assess large genetic changes and a targeted NGS panel to cover smaller, recurrently altered genetic targets. Single‐gene assays are also frequently still employed to inform timely clinical decisions for gene targets requiring rapid turnaround times (TAT), such as *FLT3*, *IDH1*, *IDH2*, and *NPM1* at the time of AML diagnosis, when the TAT of NGS does not meet clinical needs.

### Single‐gene assays

The utility of single‐gene approaches has decreased globally due to advancements in massively parallel sequencing technologies and their widespread implementation in clinical laboratories. Single‐gene assays have several disadvantages in clinical practice. First, they require more time and effort, especially when genes span several exons, such as *TP53* or *TET2*. This leads to increased costs and higher TAT.

**Figure 3:**
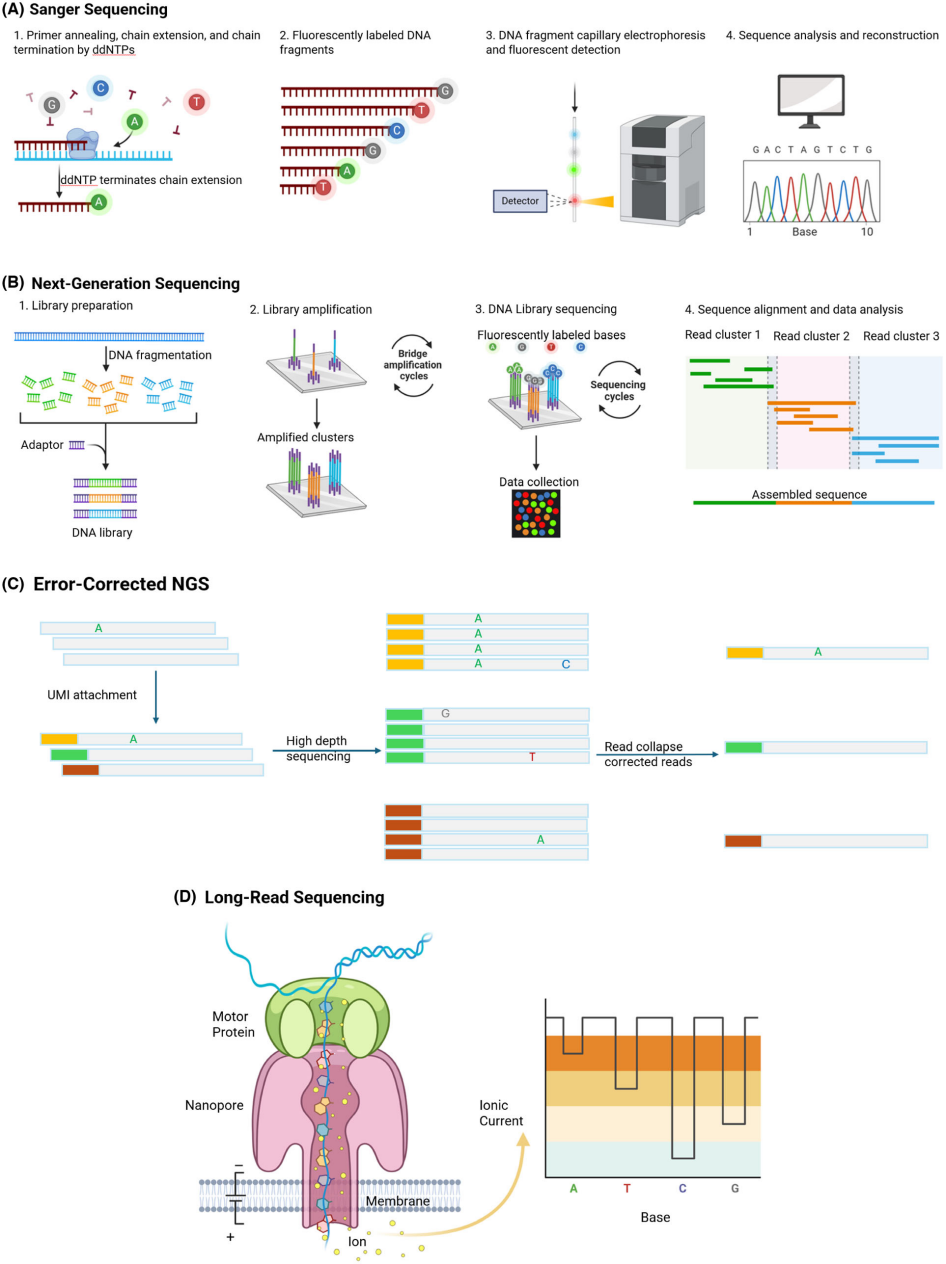
Schematic work‐flow of sequencing platforms. (**A**) Sanger sequencing is based on the selective incorporation of chain‐terminating dideoxynucleotides (ddNTPs) by DNA polymerase during DNA replication. During the chain termination PCR, DNA polymerase incorporates deoxynucleotides (dNTPs) into the growing DNA strand. When a fluorescently labelled ddNTP is incorporated, the strand elongation is terminated as ddNTPs lack a 3′‐OH group required to form a phosphodiester bond with the next nucleotide. The resulting terminated DNA fragments of various lengths are then separated by capillary electrophoresis. The fluorescence of the terminating ddNTPs is detected, and the DNA sequence is determined by the order of the fluorescence peaks recorded, which corresponds to the nucleotide sequence in the DNA strand. (**B**) Next‐generation sequencing (NGS) is a high‐throughput methodology that allows for the simultaneous sequencing of millions of DNA targets from multiple patient samples. Here, Illumina sequencing chemistry is depicted. During NGS library preparation, DNA is fragmented into smaller pieces. Sequencing adapters and indexes are ligated to the ends of these DNA fragments. The indexes enable sample identification within pooled data. Depending on whether targeted NGS or whole‐genome sequencing (WGS) is desired, a target enrichment step may be included. Libraries from multiple samples are pooled together and loaded onto a flow cell coated with oligonucleotides complementary to the sequencing adapters. The library DNA is then immobilized on the surface and undergoes bridge amplification, generating clusters of amplified DNA. These clusters augment the fluorescent signal during sequencing cycles. During sequencing, fluorescently labelled nucleotides are added. Each incorporated nucleotide emits a fluorescent signal that is detected and recorded. This process is repeated for each nucleotide in the sequence. This sequencing is performed in parallel across millions of clusters, enabling the massive parallel sequencing of multiple targets from multiple patients. (**C**) Error‐corrected NGS enhances sequencing accuracy by incorporating molecular barcodes and consensus sequence analysis to correct errors introduced during sequencing and amplification, making it especially useful in MRD assessment. During library preparation, molecular barcodes of unique molecular identifiers (UMIs) are attached to each DNA fragment before amplification. After sequencing, reads sharing the same UMIs are grouped and analysed. This allows for the identification of true mutations (e.g., “A” in all reads of the orange read group) and distinguishes them from random errors (e.g., “G”, “T”, “A” in a subset of reads in the green and red read groups). (**D**) Long‐read sequencing. Various platforms exist and Oxford Nanopore technology is depicted here. Nanopore array is submerged in an electrolyte solution and an electric potential is applied across the membrane. Double‐stranded DNA is unwound by the motor protein and the single‐strand molecule passes through the nanopore one at a time. As each nucleotide passes through the nanopore, it causes characteristic disruptions in the ionic current flowing through the pore. These disruptions generate a unique signal for each nucleotide. The device detects and records these electrical signals in real‐time, producing raw signal data that corresponds to the nucleotide sequence of the DNA molecule. A, B, and D adapted from “Sanger sequencing”, “Next generation sequencing (Illumina)”, and “Nanopore sequencing”, by BioRender.com (2024). Retrieved from https://app.biorender.com/biorender‐templates.

Furthermore, techniques like Sanger sequencing have a lower sensitivity (~20%) than NGS (3%–5%), so subclonal mutations are likely to be missed (Figure 3). Second, in complex disorders such as AML, single‐gene testing may fail to provide complete diagnostic or prognostic information. Third, single‐gene assays seldom aid in refining a differential diagnosis derived from clinical and morphological investigations; for example, when investigating unexplained cytopenia. Lastly, they cannot reveal additional, unexpected, but crucial information pertinent to patient management, such as identifying gene mutations associated with hereditary myeloid malignancy syndromes in myeloid neoplasms. That said, the use of single‐gene testing is justified in the following circumstances:

1. When single‐gene testing has a *confirmatory* role compared to clinical, morphology, and flow cytometry findings. This includes testing for *BCR::ABL1* or *PML::RARA*. Single‐gene testing is preferred here due to the rapid availability, ease of testing, and specificity of results compared to NGS. In cases of MPN where *BCR::ABL1* testing is negative, reflexive testing can be performed for *JAK2* V617F, *JAK2* exon 12, *CALR* and *MPL* gene mutations. The priority of the order of the test performed depends upon the clinical and morphological presentation.
2. When single‐gene testing has a *supporting* role compared to clinical, morphology, and flow cytometry findings. This could include *BRAF* p.V600E detection in a case that otherwise resembles HCL or *MYD88* p.L265P testing in LPL.
3. In instances where multigene testing has no clinical role. The detection of *BCR::ABL1* kinase domain mutations is an essential single‐gene investigation in CML patients with suboptimal responses to TKI, recommended by the European Leukemia Network and National Comprehensive Cancer Network.
4. As an adjunct to NGS to overcome the limitations of short‐read sequencing. One of the main drawbacks of short‐read NGS is an inability of bioinformatic algorithms to detect large (>40 bp) insertions or deletions with confidence (e.g. large *FLT3*‐internal tandem duplications). Hence, multigene myeloid panel assays should be complemented with *FLT3* fragment analysis. Similarly, a highly sensitive assay, like real‐time PCR or ddPCR, should be reflexively performed in the appropriate clinical context for *JAK2* V617F, which can occasionally present at levels below 5%, even in diagnostic cases.
5. For monitoring of disease. Real‐time PCR‐ or ddPCR‐based assays for monitoring fusion transcripts (e.g., *BCR::ABL1*, *PML::RARA*) are still the gold standard in haematological malignancies.

In resource‐constrained settings, access to genomics tests may not be readily available. In such situations, laboratories may adopt a hub‐and‐spoke model, where relatively inexpensive single‐gene tests are performed at local hospitals, and NGS testing is performed at referral laboratories. While this review focuses on molecular technologies, it is also prudent to mention the potential of considering surrogate assay choices when diagnostic yield is equivalent, or if molecular testing cannot be readily accessed. Specific protein markers in the correct pathologic context can occasionally substitute for a molecular diagnostic result, often with greater rapidity and lower cost. Such examples include immunohistochemistry (IHC) for cyclin D1 overexpression in mantle cell lymphoma, PD1 expression in clonally‐related large B‐cell lymphoma (Richter's) transformation from CLL, BRAF V600E IHC detection in HCL or certain histiocytic neoplasms, and IHC stains recognizing mutant NPM1 and CALR proteins. In other instances, the detection of a specific protein (e.g. p53 by IHC) or finding of a distinct, abnormal cytomorphologic alteration (e.g. numerous ring sideroblasts on iron stain in suspected MDS with low blasts and *SF3B1* mutation) may provide helpful “nonmolecular” diagnostic information when interpreted appropriately. Nevertheless, molecular characterization of haematologic neoplasms should be considered as a standard requirement in the current era, on account of its specificity and sensitivity. Thus, the diagnostic community should strive to ensure the dissemination of relevant expertise and capability, particularly in global regions that continue to experience logistical, economic, or technical restrictions.

### Targeted NGS assays

Targeted NGS panels (Figure 3) typically evaluate small genetic variants such as SNVs and small indels. However, depending on the assay design, they can also assess SVs and CNVs. Beyond DNA, targeted panels can also be applied to RNA, mainly for detecting recurrent translocations. In haematologic neoplasms with well‐delineated genetic landscapes, targeted panel testing offers a cost‐effective, clinically value‐based approach for evaluating gene regions of the highest clinical impact. Compared to WGS, they provide superior analytical sensitivity, faster TAT, and lower costs.^36^ Error‐correcting NGS (Figure 3) enhances deep analytic sensitivity, making measurable residual disease (MRD) monitoring possible in diseases such as AML.^37^, ^38^, ^39^

Targeted NGS testing requires a target enrichment step, which can be achieved through either hybridization capture‐based chemistry or amplicon‐based chemistry via PCR amplification. The latter tends to yield a higher percentage of reads on target, while the former offers better uniformity of gene target coverage. However, accurate variant quantification can be hindered by the percentage of PCR duplicates generated during PCR amplification. In amplicon‐based NGS, variant detection can be compromised for gene targets that are poorly amplified by PCR, e.g., a large duplication/insertion of *FLT3*‐ITD. Therefore, assay performance of various variant types and gene regions should be thoroughly validated, and assay limitations should be explicitly stated in the NGS report, with appropriate additional tests recommended. Detection of such challenging variants can be improved by using hybridization capture‐based chemistry and specialized bioinformatic tools.^36^, ^40^ Given the clinical significance of *FLT3*‐ITD, incorporating *FLT3*‐ITD fragment analysis into the AML diagnostic work‐up is a wise option to avoid missing a large *FLT3*‐ITD when using an amplicon‐based NGS panel.

### High‐throughput approaches

As a costly platform capable of comprehensively interrogating large SVs and CNVs as well as SNVs and small indels, WGS has historically been used in the molecular evaluation of constitutional diseases. However, advancements in NGS technologies have significantly reduced the cost of sequencing in recent years, allowing for a more economical and faster examination of the entire genome. A recent proof‐of‐concept study suggested a paradigm shift in the diagnostic genomic testing of myeloid neoplasms, proposing that WGS could potentially replace cytogenetic tests and even the current standard‐of‐care combination testing of cytogenetics and targeted NGS panels in the diagnostic samples.^28^ In that study, Duncavage *et al*. leveraged the relative technical simplicity of WGS, which omits the step of target enrichment necessary in the targeted panels. They sequenced the entire genome; however, focused the subsequent analysis on a whitelist of clinically relevant mutations in 40 genes and 612 SVs recurrently altered in myeloid neoplasms, and genome‐wide CNVs >5 mB. This approach was particularly informative in cases where cytogenetics were unsuccessful, with failed metaphase cultures. Compared to the targeted panel, WGS plus filtered analysis exhibited a sensitivity of 84.6% and 91.5% for SNVs and small indels, respectively. The false‐negatives were primarily attributable to low VAF or low‐coverage regions of the WGS.

Although WGS offers the potential for a unified, comprehensive genetic test for diagnosing lymphoid and/or myeloid neoplasms, its use has been limited to a few large academic institutions so far. Increased technical, bioinformatic, and analytic complexities, higher costs, longer TAT, and lack of standardization, remain significant hurdles for widespread implementation in routine clinical laboratory practice. Its successful implementation requires sophisticated infrastructure, including expertise in whole‐genome library preparation, access to high‐throughput sequencers, and specialized bioinformatics pipelines. These resources may not be readily available in many clinical laboratories and take time and effort to establish. The breadth of coverage in WGS also comes at the expense of sequencing depth, limiting its analytical sensitivity, which makes it unsuitable for e.g. AML MRD monitoring and subclonal detection. Additionally, repetitive genomic sequences present technical challenges. Short‐read sequencing approaches used in popular NGS platforms also warrant special attention to sequencing and data analysis for large genetic variants, as their detection may be compromised by low recall and high false‐positive rates. For Formalin‐fixed paraffin embedded (FFPE) samples, DNA degradation and chimeric artefacts also pose challenges and may limit its application in this sample type. Cytogenetic findings have long been the cornerstone of haematologic neoplasm diagnosis and prognosis, with their clinical value established through years of well‐powered clinico‐genetic studies. While WGS has shown potential to replace karyotyping, its added value is mostly seen in the small percentage of samples where karyotyping fails. The clinical relevance of additional findings beyond successful karyotyping still requires validation in larger studies. Furthermore, WGS performed on bulk sequencing loses the ability to reveal individual cell clones, a capability that chromosome analysis offers. Overall, cytogenetic studies remain an important, well‐established tool in haematologic neoplasms, particularly in resource‐limited settings. However, WGS is increasingly providing valuable insights, and the future may see a gradual shift toward using sequencing as the primary tool for comprehensive genomic assessments, pending improvements in cost‐effectiveness, accessibility, and standardized bioinformatics for data interpretation.

### NGS‐based assays for analysing complex IG/TR rearrangements

Over the years, IG/TR clonality assessment has been performed using different approaches. Starting with Southern blot assays, PCR‐based assays have obtained their place since the 1990s. All PCR‐based IG/TR clonality assays need to deal with the enormous and complex diversity of V, D, and J genes that are involved in these rearrangements, necessitating the use of multiple forward and reverse primers in multiplex reactions. Since obtaining an amplified PCR product *per se* does not disclose the clonal character of the rearrangements, more sophisticated readouts have been developed to distinguish identical (monoclonal) from diverse (polyclonal) PCR fragments. Of these, fragment analysis based on high‐resolution detection of differently sized, fluorescently labelled PCR fragments (also known as GeneScan analysis) has become the world standard (Figure 2).

NGS technologies, either amplicon‐based or through‐capture hybridization, have been developed and validated for assessing the clonality of B and T cell populations (Figure 2).^41^, ^42^, ^43^ These NGS approaches have the inherent advantage of not only evaluating diversity based on fragment size or nucleotide configuration, but also the actual sequence of the IG/TR rearrangement (known as the clonotype), thus allowing a more accurate assessment of clonality and even highly sensitive tracing of malignant cells during and after therapy (see below). Hence, NGS protocols will gradually take over, especially when costs go down over time. Interestingly, such IG/TR clonotype information can also be extracted from WES, WGS, or even RNA sequencing datasets.^44^, ^45^ It can be foreseen that more sophisticated single‐cell NGS analysis of the IG/TR clonotypes could become more relevant in very specific situations, such as expected intraclonal diversity.

### Optical genome mapping

OGM is a nonsequencing cytogenomic platform that has gained popularity in clinical labs in recent years. It offers a comprehensive assessment of the genome and can detect CNVs and SVs with a resolution of 5–50 kB. This is a significant improvement over the 5–10 Mb resolution for karyotyping and 100–200 kB for FISH.^46^ It utilizes ultrahigh molecular weight (UHMW) DNA molecules that are several hundred kB to mB in length. These molecules are enzymatically labelled with fluorescent tags at specific sequence motifs (CTTAAG), which are repeated throughout the genome at 4–5 kB intervals, creating a unique fluorescence pattern for each genomic region. The labelled DNA molecules are then loaded onto a chip and passed through a nanochannel array as single molecules, whose fluorescent pattern is captured, digitally analysed, and processed for genome assembly. The assembled genome is compared to a diploid reference genome, and alterations in the fluorescence pattern and label spacing are used to identify SVs and CNVs.

Multiple studies on haematologic neoplasms have demonstrated OGM as a promising alternative to standard cytogenetic platforms, as it allows for the detection of complex cytogenetic events in a single assay and reveals additional genetic events with distinct clinical significance.^47^, ^48^, ^49^, ^50^ In comparison to WGS, OGM is less costly and less complex in its implementation and analysis. However, OGM does not detect ploidy changes or provide clonal structural information and has limited ability to detect rearrangements between centromeres or telomeres, whole‐arm translocations, certain jumping translocations, and end‐to‐end telomere fusions. Also, OGM lacks sequencing capabilities and cannot detect small genetic variants (e.g. SNVs and small indels below its limit of detection). Technically, OGM requires high‐quality, UHMW DNA, which is currently only available from fresh or frozen samples (not FFPE samples). It also necessitates a dedicated DNA isolation process that differs from the routine methods used in clinical labs. These challenges add complexity to its broader implementation in nonspecialized clinical labs.

Altogether, multiple testing platforms exist, each offering unique advantages and limitations. Some newer platforms, like WGS and OGM, have the potential for platform consolidation, streamlining various types of genetic analyses into a single assay. However, the integration of these advanced technologies into routine clinical practice to replace current standard‐of‐care testing approaches depends on several critical factors: (1) clinical validation of new findings to ensure their accuracy and clinical relevance; (2) standardization of testing procedures, bioinformatic analysis, and reporting to maintain consistency across different laboratories. As these new platforms demonstrate their value through rigorous validation and standardization, they hold the promise of enhancing precision and efficiency of molecular genetic testing in clinical settings.

## Monitoring and MRD analysis

As advances in therapy continue to improve the outcomes for patients with lymphoid and myeloid neoplasms, the need for sensitive MRD assessment is increasingly important. Ideally, a neoplastic biomarker should reflect active disease status (i.e. potential for reconstituting disease relapse), be highly specific, and prognostically relevant for clinical management of the patient. Fundamental requirements of MRD assays include the demonstration of excellent analytical accuracy and precision. As target molecules approach very low concentration, analytic precision is more variable, and confident detection becomes highly dependent on the presence of a minimally sufficient amount of DNA/RNA. The impacts of efficient sample processing, high‐quality DNA or RNA extraction, and optimal time window from sample draw to testing are critical elements. In particular for RNA‐based testing, the stability of samples prior to extraction is a very important factor to minimize the effects of RNA degradation. Additional aspects, such as haemodilution of Bone marrow (BM) aspirate samples, or differential target bioanalyte bioavailability between BM and PB compartments can also have potentially significant impacts on MRD assay performance. Even with preanalytic optimization, standardization of quantitative molecular test methodologies for MRD applications is challenging. Labs with the requisite expertise may opt to develop MRD assays internally (laboratory‐developed test, LDT) and ensure operational robustness according to established guidelines (e.g. Clinical and Laboratory Standards Institute, CLSI,https://clsi.org), although commercial platform and reagent solutions are also popular options. Regardless of “build or buy” decisions, establishing and performing highly sensitive MRD assays is demanding, and includes related aspects such as the generation and maintenance of high‐quality controls and contamination mitigation strategies. Labs considering MRD test applications also require appropriate technical experience, as well as sufficient sample volume to build experience and identify potential pitfalls. Given these considerations, the issue of assay standardization is paramount to ensure interlaboratory performance quality and best patient care objectives.

In lymphoid neoplasms, MRD is mostly performed using the specific IG/TR gene rearrangements as leukaemic fingerprints. Traditionally, this has been done by designing patient‐specific primers to be used in qPCR or ddPCR.^51^, ^52^, ^53^, ^54^ With the advent of high‐throughput sequencing technologies, the field is now gradually adopting NGS as a generic approach to generate insight into the entire IG and/or TR repertoire, followed by bioinformatic analysis to trace back and quantify the patient‐specific IG/TR rearrangement.^42^ NGS‐based IG/TR MRD determination can be done using commercially available assays.^55^ or even via servicing companies, but there are also academically sourced solutions.^55^, ^56^, ^57^ The latest development concerns MRD assessment in cell‐free DNA fractions, which seems especially promising for monitoring more localized mature lymphoid neoplasms such as DLBCL.^58^ Finally, in precursor and mature lymphoid neoplasms, MRD monitoring can also be performed using SNVs or indels that are present in the respective neoplasm,^59^, ^60^ but in most instances the more widely available IG/TR fingerprints are preferred.

For myeloid neoplasms, particularly CML and AML, expressed fusion gene transcripts represent attractive targets that confer leukaemia subtype specificity and can be detected at very low quantitative abundance by qPCR techniques. Knowledge of breakpoint‐fusion genetic heterogeneity is critical for the development of comprehensive, diagnostically sensitive assays. For example, both *BCR::ABL1* and *PML::RARA* gene fusions may be associated with multiple possible chimeric mRNA transcript isoforms arising from variation in genomic break sites in one or both partner genes.^61^, ^62^ In an individual patient with leukaemia, determination of the correct fusion isoform is required to enable subsequent disease‐specific monitoring following therapy. qPCR is a widely employed methodology for expressed fusion gene product assessment but requires careful assay design, ongoing quality assurance, and sufficient control and calibration reagents to ensure optimal performance. For rare fusion gene targets, testing by clinical reference laboratories with greater sample volume experience is desirable.

## Data interpretation, data management, and bioinformatics

Molecular analysis generates complex information and potentially large datasets. The use of analytic tools, interpretive guidelines, standard reporting requirements, secure data storage, and retrieval of information represent unique aspects of the molecular testing environment. For single‐gene assays that provide a binary “positive/negative” qualitative result, analysis and reporting are relatively straightforward. More complex quantitative methods such as qPCR or ddPCR require additional evaluations of controls (calibration reagents, standard curve) and template integrity. For some quantitative PCR platforms (e.g. Roche LightCycler, Bio‐Rad QX system), vendor‐supplied analysis software provides a convenient user interface for processing assay results; however, the lab scientist or pathologist should develop proficiency and understanding of these tools and potential shortcomings to avoid interpretive mishaps. Similarly, laboratory‐developed analytic algorithms require thorough verification and benchmarking prior to clinical lab implementation. An additional complication of highly sensitive NGS methods is the identification of rare clonal haematopoietic genetic alterations that are not tumour‐related.

NGS applications generate a commensurately large amount of data, which includes relevant biologic information as well as method‐related artefacts. Rapid and ongoing proliferation of data processing and analysis tools are continuing to push the envelope of information that can be derived from NGS experiments. Collectively, management of NGS data is in the domain of bioinformatics. Bioinformatic packages may include open source such as the Genome Analysis Toolkit (GATK),^63^ developed and maintained by the Broad Institute, and the Galaxy platform.^64^, ^65^ These sources encompass numerous modules that cover data preprocessing steps (read filtering, adapter trimming, duplicate removal, read mapping to reference, base quality calibration, etc.) to produce analysis‐ready files. Data analysis is then pursued using dedicated tools to detect specific sequence variants (indels, SNVs, CNVs, and others). Specialized applications are also used to process and analyse other types of datasets (e.g. RNA sequencing, methylation, etc.). Comprehensive vended solutions are also available. Academic and commercial laboratories with strong bioinformatic core expertise may also opt to develop bespoke solutions incorporating open source and contracted products, to optimize functionality and/or flexibility. Bioinformatic pipelines are typically complex and replete with numerous filter and configuration settings that can affect the final data output. For the demands of consistency, accuracy, and reproducibility the clinical lab must therefore establish standard procedures for using and monitoring bioinformatic pipelines, including collection and review of numerous quality‐control metrics. Because of the marked proliferation of available bioinformatic tools and potential risks with implementation, expert guidance has been advanced to standardize the concepts and requirements for clinical utilization.^66^, ^67^, ^68^, ^69^, ^70^ The need for ongoing professional consensus on specific bioinformatic applications, “dry lab” or *in silico* proficiency evaluation^71^, ^72^, ^73^ and “gold standard” datasets will become even more critical as artificial intelligence (AI)‐based enhancements become incorporated into these algorithms. Secure data storage, costs, and accessibility represent new challenges. While “per base” NGS costs continue to decrease, the overall financial impact for data storage can be substantial for clinical laboratories. Cloud‐based analytics and storage solutions are widely available and are scaling rapidly but require critical regulatory oversight to ensure patient safety and privacy. Despite such challenges, the future of comprehensive molecular diagnostics must rely on robust, safe, redundant, and scalable data resources.

An ongoing challenge with data of high complexity and dimensionality concerns the manner in which information can be efficiently displayed and utilized in the clinical setting. To this end, a persistent gap in processed NGS data management is the ability to effectively communicate relevant information in clinical reports. The future of NGS testing and integrated reporting will likely need to rely on improved ways of visualizing and summarizing complex tumour genetic data in a digestible, actionable, and concise format. As the breadth and depth of information expands further, driven increasingly by genomic sequencing, incorporation of “complete” data into the clinical report (e.g. including cytogenomics, immunophenotyping results, and others) will become more consequential for optimal clinical management of patients with haematologic neoplasms.

## Intra‐ and interlaboratory quality aspects of molecular tests

Now that many molecular markers have found their way into classification and risk stratification systems of haematological neoplasms and other biomarkers are to follow, molecular testing has gained front stage in haematopathology diagnostics. As a logical consequence, the validity of molecular test results of both single‐gene assays and more complex high‐throughput assays has to be assured at both the intra‐ and interlaboratory level, which entails various quality aspects. First, for laboratories performing these tests in routine diagnostics their molecular methods have to be validated under particular quality standards (ISO, CLIA/CAP, or similar). Second, individual run performance has to be monitored, which requires internal quality controls, for which commercially available reference standards sometimes are available. Third, accuracy of test results in individual laboratories has to be assessed in external quality control schemes, which are mostly offered through officially accredited national or international organizations (such as UKNEQAS, GenQA). Additionally, organizations like the European Research Initiative on CLL (ERIC) (*TP53* mutation analysis, IGHV SHM analysis), EuroClonality (IG/TR analysis), and EuroMRD (molecular MRD testing in ALL, CML, NHL) offer laboratories the opportunity to be certified, while providing education and training programs to improve laboratory performance. Another challenge for molecular diagnostics concerns new legislation requiring laboratories to make use of IVD‐certified commercial assays, and or FDA‐ or EMA‐approved assays or services over LDTs. Of note, while the overall concept here is to improve the quality of laboratory diagnostics, it might make (molecular) assays also more costly, and thus less affordable in certain areas on the globe. LDTs might be left for those molecular markers that are being studied in a research setting, but have not been implemented as routine molecular tests.

## Conclusion and perspective

In conclusion, we are facing a true revolution concerning the implementation of molecular techniques in haematopathology diagnostics, both in lymphoid and myeloid neoplasms. These molecular methods not only impact diagnosis and classification, but are also crucial in view of prognosis, therapeutic choices, and monitoring. Although over the years molecular tests have transformed from single‐gene assays to high‐throughput methods, single‐gene assays still have their place, if only because of better accessibility and affordability in particular areas over the world. Parallel to the implementation of more complex assays, there are increasing bioinformatic challenges, which entail computational power and storage (cloud infrastructure), but also interpretation of somatic variants vs. germline variants and age‐associated clonal haematopoiesis.

It can be foreseen that in the next 5–10 years new molecular biomarkers will be discovered that would classify for implementation in routine diagnostics. Also, as technology evolves, new approaches and concepts come into reach, including other omics (e.g. epigenome, methylome, microbiome, pharmacogenomics), which will require the introduction of AI for interpretation and even report writing. Also, single‐cell assays might become implemented into routine settings, as they allow studying subclonal molecular changes as signs of emerging resistant clones or early relapses. The future for molecular diagnostics in haematopathology is bright!

## Author contributions

All authors have actively contributed to the writing of this review, as well as approved the final version.

## Conflict of interest

The authors declare that they have no conflict of interest for this review.

## Data Availability

Data sharing not applicable to this article as no datasets were generated or analysed during the current study.
